# Secretory phospholipase-A2 and fatty acid composition in oral reactive lesions: a cross-sectional study

**DOI:** 10.1186/s12935-017-0414-x

**Published:** 2017-04-27

**Authors:** Ali Hossein Mesgarzadeh, Abolfazl Akbarzadeh, Ali Rasipour, Tannaz Rasipour, Amir Mehdizadeh, Maghsod Shaaker

**Affiliations:** 1 Department of Oral and Maxillofacial Surgery, Faculty of Dentistry, Tabriz University of Medical Sciences, Tabriz, Iran; 2 Drug Applied Research Center, Tabriz University of Medical Sciences, Tabriz, Iran; 3 Liver and Gastrointestinal Diseases Research Center, Tabriz University of Medical Sciences, Tabriz, Iran; 4 Department of Biochemistry and Clinical Laboratories, Faculty of Medicine, Tabriz University of Medical Sciences, Tabriz, Iran

**Keywords:** Fatty acids, Hyperplasia, Inflammation, Phospholipase A2, Oral cavity

## Abstract

**Background:**

Oral reactive lesions are the most common lesions of oral cavity. Phospholipases and fatty acids play key roles in the creation of inflammation by change in metabolic activities and production of lipid mediators. The aim of this study was to investigate the amount of secretory phospholipase-A2 (sPLA2) and difference of fatty acid pattern in oral reactive hyperplasia and adjacent normal appearing tissues in patients with oral reactive lesions.

**Methods:**

Paired samples of oral hyperplastic (OH) and adjacent normal-appearing tissue of 45 patients were investigated in this study. The collected samples were analyzed with enzymatic spectrophotometric method in terms of the amount of sPLA2 and composition of fatty acids by gas–liquid chromatography method.

**Results:**

The amount of sPLA2 (1.8-fold, p < 0.001), stearic acid (1.2-fold, p < 0.001), oleic acid (1.1-fold, p = 0.01), arachidonic acid (1.5-fold, p < 0.001) and docosahexaenoic acid (1.3-fold, p = 0.02) were increased, while the amount of palmitoleic acid (−45%, p < 0.001) and linoleic acid (−19%, p < 0.001) were reduced in the OH tissue samples. Furthermore, the results demonstrated significant associations between the type and location of tissue samples with monounsaturated fatty acids (MUFAs) and n−3 polyunsaturated fatty acids. Tissue samples from patients with inflammatory fibroepithelial hyperplasia showed relatively higher MUFAs and lower n−3 polyunsaturated fatty acids than other type of lesions.

**Conclusions:**

Localized changes in the sPLA2 activity and composition of fatty acid are associated with oral reactive hyperplasia and the type of pathological response. We suggest that sPLA2 activity and multiple type of fatty acids might be used as potential therapeutic target for oral reactive hyperplasia.

## Background

Oral mucosa is constantly exposed to internal and external stimuli [1]. Inflammatory hyperplasia denotes a wide range of enhanced growths occurring in the oral mucosa. Oral reactive lesions are the most common lesions of oral cavity. Secretory phospholipase A2 (sPLA2) is a family of enzymes that catalyze the hydrolysis of fatty acids in the sn-2 position of glycerophospholipids of plasma membrane [2]. sPLA2 and fatty acids produce a number of key bioactive mediators such as prostaglandins and leukotrienes [3]. Both of them participate actively in the regulation of cellular gene expression by altering and activating the cell surface receptors or activating of transcription factors [3]. Clinical studies have shown that sPLA2 isoforms are increased in various cancers including oral squamous cell carcinoma [4], breast, colon, pancreas, and prostate cancers, and tonsillitis [5–8].

Emerging evidence supports the significance of sPLA2 and fatty acids as biomarkers for inflammation. However, no study has yet investigated the relationship of oral reactive lesions with sPLA2 and fatty acids so far. Therefore, this study was conducted to investigate the difference in sPLA2 and fatty acids in the oral reactive hyperplasia and adjacent normal appearing tissues, as well as their possible relationship with clinical and pathological characteristics.

## Methods

### Sampling

This cross-sectional study was done on a population of patients referred to the department of Oral and Maxillofacial Surgery at Tabriz Faculty of Dentistry to remove reactive lesions of the oral cavity. Exclusion criteria of this study were being over 70 years of age, a history of smoking, alcohol intake, inflammatory or infectious diseases or diabetes.

Paired oral hyperplastic (OH) tissue and adjacent normal-appearing tissue were taken from the same subjects to minimize the possibility of confounding factors. The protocol of this study was reviewed and approved by the ethics committee of Tabriz University of Medical Sciences and informed consent was taken from all patients. Tissue sampling was done consecutively by a single oral and maxillofacial surgeon. Both OH tissue and adjacent normal-appearing tissue (0.5 cm beyond the OH tissue) were obtained at the same time during surgery. We only collected tissue samples from localized reactive lesions in oral cavity. In all cases, presence of a local irritant was the cause of inflammation. The type of adjacent normal-appearing tissue was the same as reactive lesion and was confirmed by postoperative histology. This tissue section was used as the negative control in our study and all measures (sPLA2 and fatty acids) in reactive tissue were compared with that in adjacent normal-appearing tissue.

A total of 45 reactive tissue samples and 45 adjacent normal-appearing tissue were collected from 45 patients. All tissue samples were histologically assessed by a pathologist to confirm histopathologic status, homogeneity and integrity of the tissue. The specimens were divided and either stored at −70 °C for fatty acid analyses or snap-frozen for protein isolation.

### Secretory phospholipase A2 activity

Total protein was extracted from 20 mg of tissue specimens homogenized in ice-cold lysis buffer containing protease inhibitors, as previously described by us [9]. The suspension was centrifuged and protein concentration in the supernatant was measured using the method of Lowry [10]. The activity of serum sPLA2 was estimated by a standard assay with Diheptanoyl Thio-Phosphatidylcholine as substrate (Cayman Chemicals, Windham, NH, USA) using an Immunoscan model 310 microplate reader (Labsystems, Helsinki, Finland). Values were normalized to the corresponding total protein and expressed as units of sPLA2 activity/mg protein.

### Fatty acid analysis

Total lipids were extracted from tissue samples and esterified with methanol during catalysis with acetyl chloride [11]. Fatty acid methyl ester mixtures were separated on a 60 × 0.25-mm Teknokroma TR CN100 column using a Buck Scientific model 610 gas chromatograph (SRI Instruments, Torrance, USA) equipped with a split injector and a flame ionization detector. The oven temperature was set at 210 °C, and injector and detector temperature at 265 °C. Fatty acids were identified by comparing the peak retention time and analyzed corresponding standards (Sigma Chemicals, St. Louis, MO, USA), and were quantified by calculation the area under curve.

### Statistical analysis

Data were tested for normal distribution by histogram and Kolmogorov–Smirnov normality test. Difference in the amount of sPLA2 and fatty acids in the OH and the adjacent normal-appearing tissue was analyzed using paired t test, and an unpaired t test was used for comparing unpaired data. The significant relations between amount of sPLA2 and fatty acids and clinical variables were examined by Pearson correlation test. A p value of <0.05 was considered statistically significant. The power to detect a difference in fatty acids and sPLA2 between tissue sample groups was calculated based on the values of mean, standard deviation and number of samples for paired t test statistical analysis. All analyses were carried out using SPSS for windows version 11.0 (SPSS Inc., Chicago, IL, USA).

## Results

**Fig. 1:**
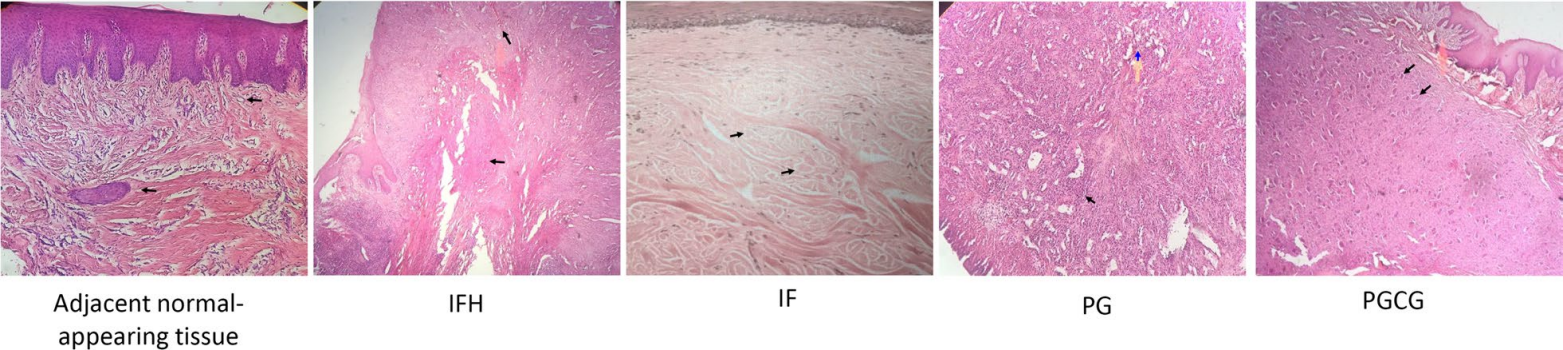
Histological sections representing the normal-appearing oral mucosa and oral reactive lesions (H&E staining ×40). *IFH* inflammatory fibroepithelial hyperplasia, *IF* irritation fibroma, *PG* pyogenic granuloma, *PGCG* peripheral giant cell lesion. *Black arrows* indicate normal fibrovascular connective tissue in adjacent oral mucosa, fibrotic connective tissue in IFH, dense collagen bundles in IF, infiltration of inflammatory cells in PG, giant cells in PGCG. *Blue arrow* indicates endothelium-lined channels in PG

This study was carried out on 45 patients in which 19 of them were male and 26 of them were female. The reactive tissues included 24 cases with inflammatory fibroepithelial hyperplasia (IFH), 10 cases with irritation fibroma (IF), 6 cases with pyogenic granuloma (PG), and 5 cases with peripheral giant cell lesion (PGCG). Representative histological sections of adjacent normal-appearing tissue and the oral reactive lesions are shown in Fig. 1. Most oral reactive lesions were observed in the anterior mandible (35%). The clinical and pathology details of the study subjects are presented in Table 1.

**Table 1:** Demographic characteristics of the 45 studied patients with oral reactive hyperplasia

Age, years^a^	47.6 ± 14.6
BMI, kg/m^2^	24.3 ± 3.85
Sex, women %	58
Type of lesion, %	
IFH	54
IF	22
PG	13
PGCG	11
Location of lesion, %	
Anterior maxilla	27
Posterior maxilla	11
Anterior mandible	35
Posterior mandible	11
Buccal mucosa	16

^a^Values are mean ± SD

Table 2 shows the level of sPLA2 quantified by enzymatic spectrophotometric assay and Table 3 presents the level of fatty acids measured by gas–liquid chromatography method in the oral hyperplastic (OH) tissue and adjacent normal-appearing tissue from the same patients with oral reactive lesions.

**Table 2:** Secretory phospholipase-A2 activity of normal-appearing and oral hyperplastic tissue

µmol/min/mg protein	Normal-appearing oral mucosa	Oral hyperplastic lesion	*p*
Secretory phospholipase-A2	1.56 ± 0.74	2.88 ± 0.88	<0.001

Values are expressed as mean ± SD. *p,* paired *t* test. *n* = 45

**Table 3:** Fatty acid composition of normal-appearing and oral hyperplastic tissue

% of total	Normal-appearing oral mucosa	Oral hyperplastic lesion	*p*
14:0 (myristic acid)	1.54 ± 0.94	1.36 ± 0.99	0.09
16:0 (palmitic acid)	43.28 ± 2.14	42.01 ± 3.27	0.05
16:1n−7 trans (trans palmitoleic acid)	0.26 ± 0.15	0.23 ± 0.11	0.26
16:1n−7 (palmitoleic acid)	2.98 ± 0.99	1.47 ± 0.82	<0.001
18:0 (stearic acid)	11.37 ± 4.23	13.42 ± 3.40	<0.001
18:1n−9 trans (elaidic acid)	0.90 ± 0.91	0.99 ± 0.66	0.55
18:1n−9 (oleic acid)	21.58 ± 4.56	23.40 ± 4.44	0.01
18:2n−6 (linoleic acid)	13.86 ± 2.92	11.27 ± 2.23	<0.001
20:0 (arachidate)	0.17 ± 0.14	0.18 ± 0.11	0.72
18:3n−9 (linolenic acid)	0.20 ± 0.12	0.18 ± 0.15	0.36
20:4n−6 (arachidonic acid)	3.21 ± 1.33	4.69 ± 1.70	<0.001
20:5n−3 (eicosapentaenoic acid)	0.35 ± 0.24	0.40 ± 0.18	0.23
22:6n−3 (docosahexaenoic acid)	0.30 ± 0.16	0.39 ± 0.21	0.02

Values are expressed as mean ± SD. *p*, paired *t* test. *n* = 45

**Fig. 2:**
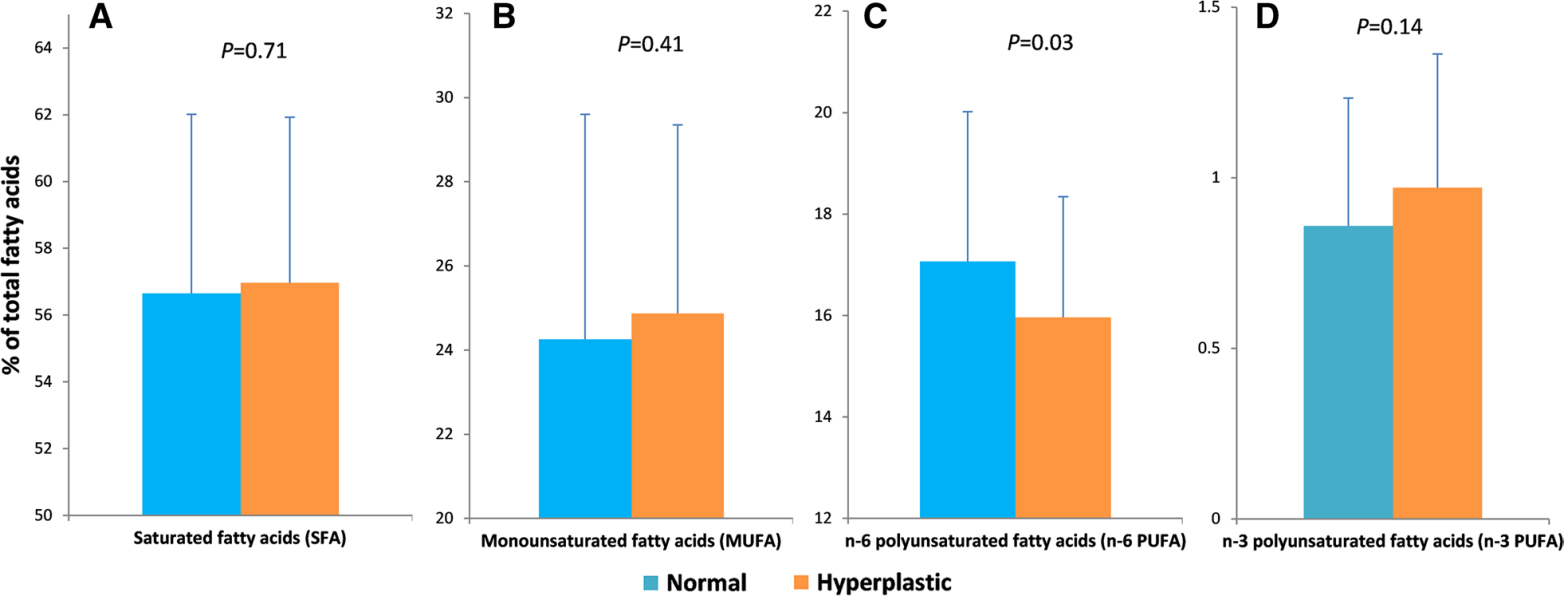
Fatty acids in normal-appearing and oral hyperplastic tissues. Mean ± SD fatty acid content (**A** saturated; **B** monounsaturated; **C** n−6 polyunsaturated; and **D** n−3 polyunsaturated fatty acids) of normal-appearing and oral hyperplastic tissues (*n* = 45)

Significant differences were found between the OH tissue samples and control tissues in sPLA2, palmitoleic acid, stearic acid, oleic acid, linoleic acid, arachidonic acid and docosahexaenoic acid. The amount of sPLA2 (1.8-fold, p < 0.001), stearic acid (1.2-fold, p < 0.001), oleic acid (1.1-fold, p = 0.01), arachidonic acid (1.5-fold, p < 0.001) and docosahexaenoic acid (1.3-fold, p = 0.02) were increased, while the amount of palmitoleic acid (−45%, p < 0.001) and linoleic acid (−19%, p < 0.001) were reduced in the OH tissue samples. In the studied patients, body mass index (BMI) was related with linoleic acid (p = 0.03, r = 0.42) of normal-appearing tissue. Among fatty acid classes, only monounsaturated fatty acids (MUFAs) were significantly different between studied tissues (p = 0.03; Fig. 2). The statistical power for detecting the reported differences, when p < 0.05, was greater than 75.3%.

**Fig. 3:**
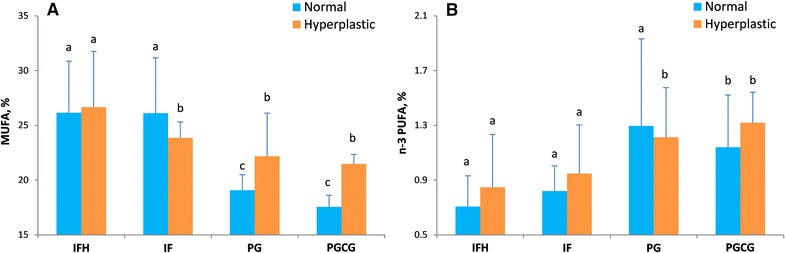
Fatty acids in different subtypes of oral hyperplastic tissues. Comparison of monounsaturated and n−3 polyunsaturated fatty acids observed according the type of oral reactive lesion and biopsied tissue sample by an analysis of variance test. *IFH* inflammatory fibroepithelial hyperplasia (*n* = 24), *IF* irritation fibroma (*n* = 10), *PG* pyogenic granuloma (*n* = 6), *PGCG* peripheral giant cell lesion (*n* = 5). *Different letters above columns* indicate significant differences among subtypes of oral hyperplastic tissues (p < 0.05)

**Fig. 4:**
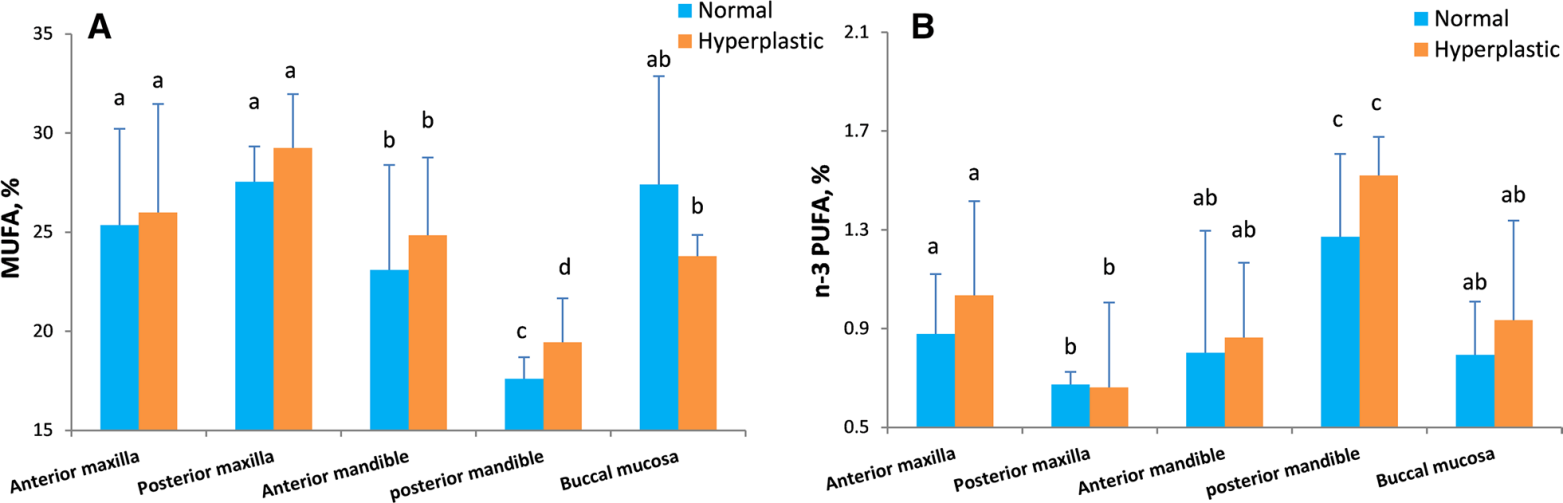
Tissue fatty acids in different oral location. Comparison of monounsaturated and n−3 polyunsaturated fatty acids observed according the location of oral reactive lesion and biopsied tissue sample by an analysis of variance test. Anterior maxilla (*n* = 12), posterior maxilla (*n* = 5), anterior mandible (*n* = 16), posterior mandible (*n* = 5), buccal mucosa (*n* = 7). *Different letters above columns* indicate significant differences among the oral locations (p < 0.05)

No association was found between the activity of sPLA2 and the type or location of the biopsies from patients with oral reactive lesion. However, sPLA2 activity showed strong significant correlations with n−6 polyunsaturated fatty acids (PUFAs) in OH tissue (r = −0.41, p < 0.001) but not in adjacent normal-appearing tissue (r = −0.03, p = 0.81). The results demonstrated significant associations between the type and location of tissue samples with total MUFA and n−3 PUFAs. Tissue samples from patients with IFH lesions showed relatively higher MUFAs and lower n−3 PUFAs than other type of lesions (Fig. 3). Posterior mandible showed the lowest amount of MUFA and the highest n−3 PUFA as compared to other locations (Fig. 4).

## Discussion

Given the high prevalence of reactive lesions of the oral cavity and altered metabolism of sPLA2 and fatty acids in the hyperplastic tissues, we investigated the hypothesis that amount of sPLA2 and composition of fatty acids in the reactive tissue and adjacent normal-appearing tissue of oral cavity will be different. The results showed that the amount of sPLA2 and arachidonic acid were increased in hyperplastic tissues. sPLA2 plays a significant role in increasing the fluidity of the cell membrane [12]. As an inflammatory agent, it promotes the inflammation through producing the prostaglandins and leukotrienes [13, 14]. The formation and growth of some tumors has been linked to sPLA2 [4, 15, 16]. Cellular phospholipids is hydrolyzed by sPLA2 and provide precursors for the production of eicosanoids [17, 18]. Eicosanoids are known as mediators of inflammatory and immune responses. Inflammatory reactions are involved in the different stages of cell proliferation [19]. Due to the increased activity of this enzyme in the reactive lesions of oral cavity, it appears that sPLA2 inhibitors can be used to treat these lesions. However, further mechanistic studies in this area are needed.

Fatty acid composition of membrane lipids could modulate several metabolic activities, such as glucose metabolism and permeability of the membrane [20]. In a study conducted by Ciҫek et al. [21], it has been shown that amount of arachidonic acid, docosahexaenoic acid, palmitoleic acid, linoleic acid and stearic acid is different in unpaired hyperplastic tissues and normal-appearing marginal tissues [21]. They have collected hyperplastic tissue and control tissue samples from different subjects. However, our samples were paired from the same subject. The difference in our findings with the above mentioned study may be due to difference in sampling methods. Since our samples was paired, it seems that there have been less confounding factors. Arachidonic acid, as an inflammatory precursor, is associated with increased inflammatory cytokines [22] and membrane fluidity [23]. Increased amount of arachidonic acid in the oral reactive lesions of oral cavity in this study was consistent with previous studies and represents a change in the lipid metabolism in hyperplastic tissues.

The results of this study showed altered levels of long chain n−3 polyunsaturated fatty acids that can be synthesized endogenously from ɑ-linolenic acid. While other studies have shown anti-inflammatory and anti-neoplastic effects of n−3 polyunsaturated fatty acids [24–26], its inhibitory effect on cancer cells is under intense investigation [27]. Similar to our data, a recent study has indicated an increase of docosahexaenoic acid in the reactive tissues of oral cavity [21]. Current evidence indicate that inflammatory response influence tissue turnover and availability of docosahexaenoic acid [28]. Notably, nonsurgical periodontal treatment significantly increased the serum levels of n−3 PUFAs [29]. Furthermore, the relative content of n−3 PUFAs in membrane phospholipids is higher than in the total cellular lipids. Increased ratio of cellular membrane to tissue volume is a main characteristic of oral hyperplastic and oral cancer tissues. Therefore, it seems that membrane phospholipids are more important in determining the content of DHA in exophytic tissues like oral hyperplasia and oral cancers than in normal margins.

According to the literature, while n−3 polyunsaturated fatty acids have anti-inflammatory effect, n−6 polyunsaturated fatty acids have mainly inflammatory effects [30]. In this study, OH tissue had significantly lower n−6 PUFAs than adjacent control tissue. This decrease may be due to higher consumption of these fatty acids in eicosanoid pathway. This finding could also be indicative of specific changes in different fatty acids.

Studies have shown that saturated fatty acids (SFAs) cause inflammatory response and their high intake is associated with periodontal disease [31]. In the current study, although total SFA showed no changes, stearic acid was higher in OH tissue compared to control tissue. Metabolic importance of each SFA is different. Indeed, induction of inflammation and apoptosis is not the same among different saturated fatty acids [32, 33].

Fatty acids in each group of SFA, MUFA, n−3 PUFA and n−6 PUFA have properties close to each other. However, it seems that contrary to previous studies where changes in each of these categories of fatty acids is proposed as a diagnosis biomarker, weak or no associations were found between overall amount of these groups and reactive tissues of oral cavity. Instead, each fatty acids showed different and unique association with oral reactive lesions. In a previous study, similar variation trends of sPLA2 and other fatty acids has been reported in oral squamous cell carcinoma [4]. Both malignant and oral reactive lesions are exophytic tissues with inflammatory and hyperplastic presentations. Accordingly, our data show notable similarity between oral cancers and oral reactive tissues in variations of sPLA2 and some fatty acids as major inflammatory factors. Differential diagnosis of oral squamous cell carcinoma and oral hyperplasia is currently based on clinical and pathology examination. It is remained to be investigated in future studies whether there is a difference between oral squamous cell carcinoma and oral hyperplasia in sPLA2 and tissue fatty acids.

Inflammation is heterogeneous in terms of pathogenesis and cellular molecular events. Reactive lesions in oral cavity are characterized by a severe localized inflammation. This inflammation is an immune response that is triggered by localized stimuli, such as mechanical stress and microtrauma, and infections. sPLA2 and cellular fatty acids play a crucial role in inflammatory processes. Inflammatory cytokines can modulate several enzymes related to fatty acid synthesis and degradation. According to our data, BMI of patients was associated with the amount of linoleic acid in control tissue. High BMI is a risk factor for many diseases [34]. Epidemiologic studies show gradual increase in dietary intake of linoleic acid worldwide due to nutritional transitions which has been linked to the development of obesity [35]. As linoleic acid is a precursor of inflammatory mediators, it could be suggested that this n−6 PUFA is a link between BMI and systemic inflammation which is associated with increased risk of inflammatory disorders. In consistent with this hypothesis our results show a significantly lower levels of linoleic acid in OH tissue, possibly due to its local consumption for production of inflammatory mediators.

Our findings showed that the pathologic characteristics and location of oral reactive hyperplasia determine the pattern of fatty acids. MUFAs are major plasma fatty acids. Therefore, increased total MUFA in IF and IFH may be due to higher access to blood circulation rather than a result of endogenous metabolic changes. In contrast, the activity of desaturases may negatively be affected in the PG and PGCG lesions. These enzymes endogenously produce MUFAs from cellular saturated fatty acids.

A major advantage of this study was direct investigation of paired samples collected from the same patients without any systemic inflammation. However, the number of subjects in the subgroups based on the type of pathological response is relatively small to make definitive conclusions. Moreover, it has been shown that composition of the dietary fatty acids influence tissue fatty acids. Future studies with larger sample size, and more homogenous populations in terms of diet and gender, and analysis of enzymes related to fatty acid synthesis and degradation would be helpful.

## Conclusions

It is concluded that localized changes in the sPLA2 activity and composition of fatty acid are associated with oral reactive hyperplasia and the type of pathological response. We suggest that sPLA2 activity and multiple type of fatty acids might be used as potential therapeutic targets for oral reactive hyperplasia.

## Abbreviations

sPLA2: secretory phospholipase-A2
SFA: saturated fatty acid
MUFA: monounsaturated fatty acid
PUFA: polyunsaturated fatty acid
BMI: body mass index
OH: oral hyperplastic
IFH: inflammatory fibroepithelial hyperplasia
IF: irritation fibroma
PG: pyogenic granuloma
PGCG: peripheral giant cell lesion
